# Supplementary material for: Altered microRNA Transcriptome in Cultured Human Liver Cells upon Infection with Ebola Virus
Source: Int J Mol Sci. 2021 Apr 6;22(7):3792. doi: 10.3390/ijms22073792 (PMC8038836; doi:10.3390/ijms22073792)
Supplement: Supplementary file 1 [file ijms-22-03792-s001.zip › Supplementary_File/C_ GO_Analysis_Results/16-30nt_go_Mayinga-96h-Huh7_vs_Control-96h-Huh7_down.mature_mirna_targets/CC_FoldEnrichment.pdf]

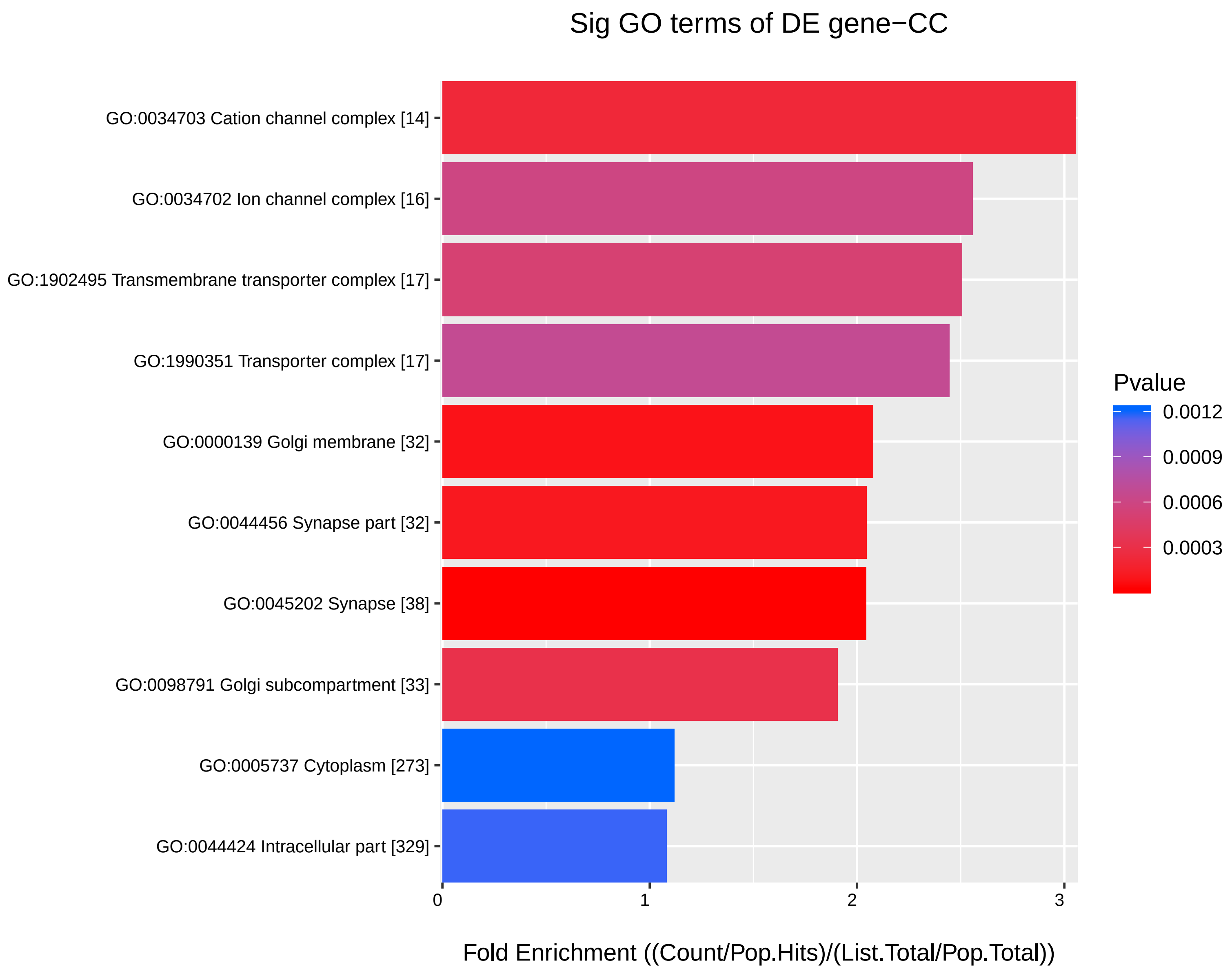
Sig GO terms of DE gene−CC
GO:0034703 Cation channel complex [14]
GO:0034702 Ion channel complex [16]
GO:1902495 Transmembrane transporter complex [17]
GO:1990351 Transporter complex [17]
GO:0000139 Golgi membrane [32]
GO:0044456 Synapse part [32]
GO:0045202 Synapse [38]
GO:0098791 Golgi subcompartment [33]
GO:0005737 Cytoplasm [273]
GO:0044424 Intracellular part [329]
0
1
2
3
Fold Enrichment ((Count/Pop.Hits)/(List.Total/Pop.Total))
Pvalue
0.0012
0.0009
0.0006
0.0003
